# Contributions of Cognitive-Motivational Factors to the Sense of Identity

**DOI:** 10.1007/s12144-016-9435-1

**Published:** 2016-03-23

**Authors:** Aleksandra Pilarska

**Affiliations:** Department of Personality Psychology, Institute of Psychology, Adam Mickiewicz University, Szamarzewskiego 89, 60-568 Poznań, Poland

**Keywords:** Cognitive-motivational dispositions, Sense of identity, Gender differences

## Abstract

This study addressed the relationship between sense of personal identity and thinking dispositions such as need for cognition, reflection, and integrative self-knowledge as well as modes of coping with self-related discrepancies through either excessive assimilation or accommodation. Participants were 544 young adults. The correlation and path analyses revealed, as expected, that need for cognition and integrative self-knowledge positively influenced one’s sense of identity, while over-responsiveness to discrepant information about the self influenced it negatively. The effects of reflection and imperviousness to discrepancies appeared more complicated and varied. Together, the findings confirm the importance of cognitive-motivational variables in the development and maintenance of a sense of identity, and suggest that gender differences in their relative significance may deserve additional research attention.

## Introduction

The notion that the identity formation process requires certain cognitive capacities is well-established in psychological literature (e.g., Hilgard 1949; Inhelder and Piaget 1958; Maruszewski 2010). Erikson himself (Erikson 1968) noted the important role cognitive processes, such as introspection, insight, and reality testing, play in identity formation. Based mainly on the work of Piaget (1977) and Erikson (1968), a number of authors have made propositions about how identity formation and maintenance relates to cognitive functioning (e.g., Berzonsky 1991; Bosma and Kunnen 2001; Breakwell 1993; Grotevant 1987; Kerpelman et al. 1997; Whitbourne 1986). Moreover, several studies directly linked identity development and outcomes with individual differences in cognitive functioning (e.g., Klaczynski et al. 1998; Neimeyer and Metzler 1994; Rowe 1980).

Drawing upon the cognitive-processing perspective, this study’s purpose was to propose and test a more comprehensive model of cognitive-motivational mechanisms as explanatory factors of a sense of personal identity. The investigation thus attempts to extend the study of cognitive determinants of personal identity by following the motivated cognition perspective (i.e., how people’s reasoning is affected by their motives to arrive at particular outcomes and to adopt particular strategies of information processing). The model identifies several cognitive-motivational characteristics that might be relevant to forming and maintaining a sense of identity, i.e., need for cognition, reflection, integrative self-knowledge, and imperviousness (excessive assimilation) and over-responsiveness (excessive accommodation) to discrepant experiences or information about the self. Among above listed dispositions, need for cognition is considered as non-specific, in the sense that it is not specifically aimed towards a certain object, as opposed to the remaining ones, for which the object is the self.

### Sense of Identity

Although current theories of identity (e.g., Berzonsky 1991; Luyckx et al. 2007; McAdams 1996; Vignoles et al. 2006) have gone beyond Eriksonian thinking, they all seem to agree with the spirit of Erikson’s (1980) work that a primary function of identity is to provide one with a sense of inner coherence and continuity. For this research, the sense of personal identity is defined as a comprehensive, intuitive and reflective stance toward oneself. It corresponds to the recurring modes of experiencing oneself-as-subject and is co-determined by the conscious self-representations, those that an individual considers most representative of oneself and most important (Pilarska 2014; Pilarska and Suchańska 2013a). This view is consistent with the assumption that self-identification can be attained in two ways: by experiencing oneself in relation to the world and other people, and by interpreting and reasoning from these experiences towards explicit self-descriptions (Epstein 2003; Trzebińska 2002). Such understood sense of identity is a multifaceted phenomenon comprising several identity-related senses: sense of having inner contents (thoughts, feelings, etc.), sense of uniqueness, sense of one’s own boundaries, sense of coherence, sense of continuity over time, and sense of self-worth (e.g., Erikson 1968, 1980; Oleś 2008; Pilarska 2014; Sokolik 1996; Vignoles et al. 2006). The healthy and mature sense of identity requires the development and maintenance of all of them (Sokolik 1996).

### Need for Cognition

Need for cognition is a personality construct that concerns dispositional differences in cognitive motivation. It refers to individuals’ tendencies to engage in and enjoy effortful cognitive activity (Cacioppo and Petty 1982). High-need-for-cognition individuals “naturally tend to seek, acquire, think about, and reflect back on information to make sense of stimuli, relationships, and events in their world”, whereas low-need-for-cognition individuals have little motivation for cognitively effortful tasks, and prefer to rely on others’ judgments and cognitive heuristics (Cacioppo et al. 1996, p. 198).

Many studies demonstrated that differences in need for cognition are associated with information-processing and problem-solving strategies (e.g., Cacioppo and Petty 1982; Cacioppo et al. 1983; Haddock et al. 2008; Ruiter et al. 2004; Verplanken et al. 1992) as well as with personality traits, including the Big Five (e.g., Fleischhauer et al. 2010; Sadowski and Cogburn 1997; see, for review, Cacioppo et al. 1996). Particularly noteworthy are findings suggesting that need for cognition contributes to coherence across different areas of functioning. High-need-for-cognition individuals tend to have more resistant attitudes (Haugtvedt and Petty 1992), exhibit stronger attitude-behavior consistency (Cacioppo et al. 1986), and are more able to resolve conflict and reconcile contradictory information about attitude objects (Thompson et al. 1995).

The cognitive skills used by those high in need for cognition would be especially helpful in constructing identity. Accordingly, research showed that high-need-for-cognition individuals are more likely to be at a higher level of identity development, assessed in terms of Berzonsky’s identity styles, Marcia’s identity statuses, and resolution of identity crisis as defined by Erikson (Berzonsky and Sullivan 1992; Njus and Johnson 2008), and they report greater self-concept clarity (Campbell et al. 1996).

### Reflection

Dispositional self-consciousness, an enduring tendency to direct attention toward oneself, has been of ongoing interest since Fenigstein et al. (1975) developed the Self-Consciousness Scale. However, the overall pattern of findings relating self-awareness to psychological adjustment is ambiguous – a phenomenon referred to as the self-absorption paradox or sadder-but-wiser effect (Alloy and Abramson 1979; Fleckhammer 2004; Trapnell and Campbell 1999). To reconcile the conflicting findings, several authors differentiated between functional and dysfunctional forms of self-consciousness (e.g., Anderson et al. 1996; Burnkrant and Page 1984; Martin and Debus 1999; Mittal and Balasubramanian 1987). In a similar vein, Trapnell and Campbell (1999) proposed two motivationally distinct dispositions confounded in private self-consciousness, i.e., rumination (neurotically motivated) and reflection (motivated by self-curiosity and search for self-knowledge). Numerous studies supported this proposition and confirmed reflection as a more adaptive form of self-focus (e.g., Joireman et al. 2002; Harrington and Loffredo 2010; Luyckx et al. 2007; Luyckx et al. 2008; Thomsen et al. 2011). However, its effects are not solely positive ones (Elliott and Coker 2008; Takano and Tanno 2009).

The definition of reflection alone suggests its relevance to identity formation. Indeed, previous studies found it positively associated with need for self-knowledge (Trapnell and Campbell 1999) and adaptive identity processes (Johnson and Nozick 2011; Luyckx et al. 2007; Luyckx et al. 2008). Still, there is some controversy since reflection was found negatively related to self-concept clarity (Campbell et al. 1996; Csank and Conway 2004; Johnson and Nozick 2011).

### Integrative Self-Knowledge

Proceeding on the assumption that self-knowledge is a process organized in time, Ghorbani et al. (2008) conceptualized integrative self-knowledge “as an adaptive and empowering attempt of the self to understand its experience across time to achieve desired outcomes” (p. 397). Research supports the benefits of integrative self-knowledge in enhancing mood and well-being while decreasing anxiety, depression, and various stress indicators (Ghorbani et al. 2008, 2013a, b); and relates integrative self-knowledge to broad personality traits such as the Big Five (Tahmasb et al. 2008).

It would be obvious to consider the constitutive relevance of integrative self-knowledge for the identity formation process. After all, countless theorists have pointed out that to have an identity is to achieve a sense of continuity over time (e.g., Erikson 1968; Goth et al. 2012; Sokolik 1996; Vignoles et al. 2006). Also, a multitude of research in narrative theory has emphasized that identity difficulties often result from one’s inability to construct a life story that makes sense – integrates interpretations of the past with the present self and provides life with purpose (e.g., McAdams 1996; McLean 2008; Singer 2004). Studies directly examining the effects of integrative self-knowledge on identity are scarce, but the available evidence indicates a positive correlation between integrative self-knowledge and Eriksonian measure of identity (Ghorbani et al. 2010).

### Assimilative and Accommodative Coping

Piaget’s (1977) theory includes assimilation and accommodation as the fundamental and complementary processes guiding cognitive development. Several identity models apply the concepts of assimilation and accommodation to describe processing of self-relevant information (e.g., Bosma and Kunnen 2001; Breakwell 1993; Grotevant 1987; Whitbourne 1986). Identity assimilation refers to interpretation of self-relevant events in terms of the cognitive-affective schemata that are presently held about the self. To Whitbourne, identity assimilation is primarily a defensive strategy that protects one’s sense of inner consistency and worth (Whitbourne 1986). Identity accommodation is the process of changing identity in response to experiences that are discrepant with existing self-schemata (Kunnen et al. 2008; Whitbourne 1986). Since equilibration needs both assimilation and accommodation, the utilization of either process, to the exclusion of the other, is likely to lead to maladjustment (Whitbourne 1986, 1996). The two modes of coping recognized in this paper, imperviousness and over-responsiveness, each corresponds to such extremes of assimilative and accommodative processes. Finding a balance between assimilation and accommodation is the optimal approach to handling identity concerns. Identity-balanced individuals are flexible enough to acknowledge and integrate changes within the self, yet structured enough to support their core self-conceptions and preserve their identity from disintegration (Sneed and Whitbourne 2003; Whitbourne et al. 2002).

Prior research on identity processes showed a positive link between identity balance and adaptive psychological functioning, whereas the opposite was found for identity accommodation (Polverino 2010; Sneed and Whitbourne 2003; Westerhof et al. 2012; Whitbourne et al. 2002). Less consistent associations were found for identity assimilation (Skultety and Whitbourne 2004; Westerhof et al. 2012; Whitbourne and Collins 1998).

### Summary and Aim

The cognitive-processing perspective recognizes identity formation as the process of collecting, evaluating, and organizing information about the self (e.g., Berzonsky 1991; Grotevant 1987; Schwartz et al. 2005b). The current study focuses on three cognitive-motivational variables that are known to promote meaning and integration of self-knowledge: need for cognition, reflection, and integrative self-knowledge. Each of these dispositions was positively linked psychological adjustment (Cacioppo et al. 1996; Ghorbani et al. 2008; Trapnell and Campbell 1999). Two modes of reacting to self-related discrepancies, which represent excessive forms of assimilation and accommodation, and may result in the sort of biased processing (Whitbourne 1986, 1996) – imperviousness and over-responsiveness – are also included in the analysis. The papers aims at describing how these factors might contribute in strengthening or weakening a person’s sense of identity.

Specifically, it was hypothesized that higher need for cognition would predict increased reflection (H1) and integrative self-knowledge (H2), and that reflection and integrative self-knowledge would be positively related to each other (H3). Positive associations between need for cognition, reflection, and integrative self-knowledge were previously reported by others (Ghorbani et al. 2008, 2013a, b; Trapnell and Campbell 1999). It was also expected that those low in need for cognition would be more likely to be imbalanced in terms of assimilation (H4) and accommodation (H5). The overuse of assimilation is accompanied by lack of insight and defensive rigidity, and the overuse of accommodation is marked by reliance on others for self knowledge and direction (Kroger 2002; Whitbourne 1986; Whitbourne et al. 2002) – all of which are characteristic of low-need-for-cognition individuals (Cacioppo et al. 1996; Fletcher et al. 1986; Ghorbani et al. 2004).

Both, imperviousness and over-responsiveness were predicted to have negative impact on reflection (H6 and H7, respectively) and on integrative self-knowledge (H8 and H9, respectively). Those highly impervious to change (i.e., assimilators) lack motivation to engage in introspection or self-analysis because they do not value the outcomes nor do they enjoy the process (Whitbourne 1986). The predicted negative relationship of over-responsiveness with reflection is more tenuous. The loss of inner integrity in excessive accommodators leads to self-doubt and low self-esteem, and can foster self-focus, but it would probably take the form of neurotic rumination rather than inquisitive reflection (Whitbourne 1986; Sneed and Whitbourne 2003). However, the two types of self-focus tend to be positively correlated (Luyckx et al. 2007; Takano and Tanno 2009; Trapnell and Campbell 1999).

Based on previous findings (Berzonsky and Luyckx 2008; Berzonsky and Sullivan 1992; Ghorbani et al. 2008; Johnson and Nozick 2011; Luyckx et al. 2007; Luyckx et al. 2008; Njus and Johnson 2008), higher need for cognition, higher reflection, and higher integrative self-knowledge each was expected to contribute positively to sense of identity (H10, H11, and H12, respectively). Such cognitive engagement should enable individuals to be more open to self-experience, more active in seeking and evaluating self-relevant information, and more efficient in processing information about the self, thereby more likely to resolve discrepancies within the self-concept and to integrate self-relevant knowledge into a meaningful whole. In keeping with identity process theory (Whitbourne 1986; Whitbourne et al. 2002), which suggests that identity accommodation is associated with a diffuse and unstable identity, over-responsiveness was hypothesized to be negatively predictive of sense of identity (H13). Conversely, since identity assimilation provides escape from negative feelings about one’s self and serves a protective function in preserving one’s sense of continuity over time, imperviousness was expected to be positively predictive of sense of identity (H14). However, given the defensive nature of excessive assimilation, the latter hypothesis should be considered as less certain.

## Method

### Participants and Procedure

The data was collected as part of a larger research project. The sample included 544 Polish students (59.4 % female) of different faculties and education institutions (universities and higher professional schools), whose age ranged from 18 to 32 years (*M* = 21.29, *SD* = 1.46). Participants were informed about the research’s purpose and were ensured about anonymity and confidentiality. They completed measures of identity and thinking dispositions, along with other questionnaires not relevant to this study.

### Measures

#### Sense of Identity

To measure sense of personal identity extended form of the Multidimensional Questionnaire of Identity^1^ (MQI; Pilarska 2012, 2014) was employed. This 45-item questionnaire consists of six subscales measuring the strength of six identity-related senses (of having inner contents, uniqueness, one’s own boundaries, coherence, continuity over time, and self-worth). All items (e.g., I feel that I was once a very different person than I am now; It happens that I perceive my close one as an important part of my self) are evaluated on a 4-point scale from “strongly disagree/never” to “strongly agree/always”. In addition, the global score (GSI) was computed by averaging scores across six subscales.^2^ For this study’s sample, the Cronbach’s alpha coefficient for the overall scale was .80, and ranged from .60 to .81 (average, .71) for individual subscales.

#### Need for Cognition

Need for cognition was assessed via an adapted version of the Need for Cognition Scale (NCS; Cacioppo and Petty 1982; Matusz et al. 2011). The scale includes 36 items focusing on engagement in and enjoyment of intellectual activities (e.g., I try to avoid situations that require intensive thinking from me; I enjoy broadening my knowledge about things); each evaluated on a 5-point scale from “strongly disagree” to “strongly agree”. In the present sample, the Cronbach’s reliability coefficient of the NCS was α = .88.

#### Reflection

Reflection, an openness-related form of self-focus, was measured with the 8-item Reflection subscale of the Rumination-Reflection Questionnaire – Shortform (RRQ Shortforms; Trapnell 1997; translated version by Pilarska and Suchańska 2013b). Every item (e.g., I love exploring my “inner” self) is presented on a 5-point scale, allowing for a range of responses from “strongly disagree” to “strongly agree”. In the current sample the Cronbach’s alpha coefficient was .80.

#### Integrative Self-Knowledge

The Integrative Self-Knowledge Scale (ISK; Ghorbani et al. 2008; Polish adaptation by Pilarska 2016) includes 12 items referring to an individual’s efforts (1) to understand past experience (e.g., If I need to, I can reflect about myself and clearly understand the feelings and attitudes behind my past behaviors), (2) to maintain awareness of the self in the present (e.g., Most of the time, I get so involved in what is going on that I really cannot see how I am responding to a situation), and (3) to move toward desired goals in the future (e.g., By thinking deeply about myself, I can discover what I really want in life and how I might get it). Each item is rated on a 5-point Likert scale, ranging from “largely untrue” to “largely true”. In this sample, the Cronbach’s reliability coefficient of the ISK was .80.

#### Coping with Self-Related Discrepancies

The Coping With Discrepancies Scale (CWD) by Pilarska and Suchańska (2013b) was used for measuring the two modes of responding to discrepant information about the self. This tool consists of 15 yes-or-no statements. Imperviousness items (8 in total) focus on attempts to minimize or deny the significance of a self-related discrepancy (e.g., After behaving contrary to my principles, I try to erase it from my memory as soon as possible; How other people see me, does not particularly interest me). Over-responsiveness items (7 in total) indicate that a discrepancy has led to anxiety and self-doubt (e.g., When I find myself in a new situation, I feel anxious that it may undermine what I think about myself; When I see how differently I react to various situation, I feel confusion and uncertainty). For this study, the Cronbach’s alpha coefficients for the two subscales were moderate (but were the best that could be obtained from this data): α = .64 for imperviousness and α = .52 for over-responsiveness.

## Results

The basic statistical description of all variables is presented in Table 1.^3^ The levels of skewness and kurtosis exhibited by the data were below those that Kline (1998) specifies as problematic (skewness: −0.40 to 0.62; kurtosis: −0.45 to 0.52). Gender differences were examined by means of U Mann-Whitney tests. As shown in Table 1, men received higher scores on reflection and imperviousness to discrepant self-relevant information. Moreover, men, as compared to women, had higher scores on sense of uniqueness, their own boundaries, and self-worth. In terms of other variables, no significant gender differences were observed.^4^

**Table 1:** Descriptive Statistics and Gender Differences for all Measures

Variable	Total sample		Women		Men		*U*	Z	Effect size *r*
	*M*	*SD*	*M*	*SD*	*M*	*SD*			
NC	127.65	17.45	127.55	17.13	127.79	17.94	34,348.50	-0.30	.01
REF	3.26	0.77	3.21	0.76	3.34	0.77	31,578.00*	-2.12	.09
ISK	2.36	0.62	2.31	0.61	2.43	0.64	31,988.00	-1.72	.07
IP	0.31	0.24	0.28	0.23	0.34	0.26	29,899.00**	-2.74	.12
OR	0.39	0.24	0.40	0.25	0.37	0.23	32,332.50	-1.28	.06
SIC	2.16	0.49	2.16	0.47	2.16	0.53	34,338.00	-0.16	.01
SU	1.72	0.49	1.66	0.50	1.79	0.46	28,724.50***	-3.25	.14
SOB	1.48	0.42	1.45	0.42	1.54	0.42	29,606.50*	-2.33	.10
SC	1.91	0.45	1.92	0.42	1.90	0.48	33,719.50	-0.57	.02
SCT	1.88	0.40	1.88	0.38	1.88	0.43	33,643.50	-0.40	.02
SSW	1.97	0.47	1.93	0.46	2.02	0.48	30,485.00*	-2.27	.10
GSI	1.85	0.32	1.83	0.30	1.89	0.35	31,474.00	-1.64	.07

*NC* = need for cognition, REF = reflection, ISK = integrative self-knowledge, IP = imperviousness, OR = over-responsiveness, SIC = sense of having inner contents, SU = sense of uniqueness, SOB = sense of one’s own boundaries, SC = sense of coherence, SCT = sense of continuity over time, SSW = sense of self-worth, GSI = global sense of identity. ***p < .001, **p < .01, *p < .05

Table 2 contains zero-order correlations between cognitive-motivational dispositions and senses of identity for the total sample and for men and women separately. The obtained results are generally in line with expectations. Need for cognition, reflection, and integrative self-knowledge were all positively correlated with one another. The two modes of responding to discrepant information about the self were negatively associated with measures of active cognitive processing, except for the correlation between over-responsiveness and reflection. Expectedly, the two modes of coping were inversely related to each other. There were no significant gender differences between these correlation coefficients.

**Table 2:** Correlation Matrix of Measures of Cognitive-Motivational Dispositions and Identity

Variable	1	2	3	4	5	6	7	8	9	10	11	12
1. NC	–	.34***	.41***	-.20***	-.33***	.29***	.30***	.10*	.31***	0.27***	0.38***	0.40***
2. REF	.31*** (.39***)	–	.27***	-.32***	-.04	-.04	.23***	.01	-.08	-.04	.10*	.04
3. ISK	.36*** (.47***)	.21*** (.35***)	–	-.26***	-.33***	.40***	.26***	.23***	.40***	.29***	.40***	.47***
4. IP	-.21*** (−.18**)	-.34*** (−.33***)	-.28*** (−.28***)	–	-.19***	.00	.04	.18***	.00	-.04	.00	.05
5. OR	-.31*** (−.36***)	-.02 (−.06)	-.30*** (−.37***)	-.22*** (−.14*)	–	-.41***	-.24***	-.26***	-.40***	-.32***	-.40***	-.49***
6. SIC	.25*** (.34***)	-.09 (.03)	.36*** (.44***)	.08 (−.09)	-.41*** (−.43***)	–	.29***	.20***	.78***	.59***	.57***	.83***
7. SU	.32*** (.27***)	.18*** (.29***)	.21*** (.33***)	.12* (−.11)	-.23*** (−.25***)	.22*** (.40***)	–	.10*	.22***	.26***	.51***	.58***
8. SOB	.11 (.10)	.01 (−.01)	.24*** (.20**)	.13* (.21**)	-.21*** (−.33***)	.21*** (.19**)	.12* (.02)	–	.31***	.04	.26***	.44***
9. SC	.29*** (.34***)	-.14* (.00)	.40*** (.41***)	.09 (−.09)	-.41*** (−.39***)	.78*** (.78***)	.16** (.33***)	.33*** (.29***)	–	.57***	.56***	.82***
10. SCT	.22*** (.32***)	-.09 (.04)	.23*** (.37***)	-.02 (−.07)	-.31*** (−.35***)	.57*** (.62***)	.15** (.43***)	.00 (.10)	.54*** (.61***)	–	.48***	.69***
11. SSW	.42*** (.34***)	.07 (.12)	.36*** (.44***)	.08 (−.13)	-.43*** (−.33***)	.53*** (.64***)	.48*** (.54***)	.32*** (.17*)	.50*** (.65***)	.40*** (.59***)	–	.81***
12. GSI	.40*** (.40***)	-.02 (.10)	.44*** (.50***)	.13* (−.07)	-.49*** (−.49***)	.81*** (.85***)	.56*** (.62***)	.48*** (.39***)	.80*** (.86***)	.63*** (.77***)	.80*** (.83***)	–

*NC* = need for cognition, REF = reflection, ISK = integrative self-knowledge, IP = imperviousness, OR = over-responsiveness, SIC = sense of having inner contents, SU = sense of uniqueness, SOB = sense of one’s own boundaries, SC = sense of coherence, SCT = sense of continuity over time, SSW = sense of self-worth, GSI = global sense of identity. ***p ≤ .001, **p < .01, *p < .05. Correlations for the total sample are presented above the diagonal; below the diagonal are correlations obtained for women and men (correlations for men are in parentheses)

Global sense of identity showed positive associations with need for cognition and integrative self-knowledge; and a negative association with over-responsiveness. The positive correlation between global sense of identity and imperviousness reached significance only for women. Fisher’s z-test indicated that this association was significantly stronger in women than in men (*z* = 2.22, *p* = .026).

Need for cognition correlated positively with all specific identity senses. Small positive associations emerged between reflection and senses of uniqueness and self-worth. The latter association was not reliable (although in the predicted direction) for women or men alone. For women only, reflection was negatively associated with sense of coherence. Integrative self-knowledge was consistently positively related to all specific identity senses. None of the above correlations were significantly different for women and for men.

A consistent pattern of negative correlations was found between over-responsiveness to discrepant self-relevant information and all specific identity senses. The overall associations between imperviousness and specific identity senses were small and reached significance only for sense of one’s own boundaries. However, patterns of association differed for men and women. There was a trend among women for imperviousness to be associated positively with specific identity senses, although the correlation attained significance only for senses of uniqueness and one’s own boundaries. With the exception of the positive correlation with sense of one’s own boundaries, imperviousness in men tended to be negatively (although not significantly) related to specific identity senses. Fisher’s z-test showed that the relationships of imperviousness with senses of having inner contents, uniqueness, coherence, and self-worth were significantly different for males and females (*z* = 1.99, *p* = .047; *z* = 2.61, *p* = .009; *z* = 2.01, *p* = .045; and *z* = 2.41, *p* = .016, respectively).

**Fig. 1:**
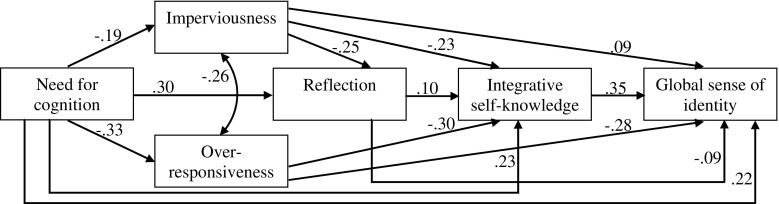
The final model. *N* = 544. Standardized path coefficients are shown; all these coefficients were significant at *p* < .05 or less

Structural equation modeling was used to further examine relationships between cognitive-motivational factors and global sense of identity. The model estimation was performed with maximum likelihood estimation. The error terms associated with the two subscales of the Coping With Discrepancies Scale were allowed to covary. The findings revealed that the over-responsiveness/reflection path was insignificant and was deleted from the model. The lack of significant effect of over-responsiveness on reflection replicated the correlation results, and supported the reasoning outlined in the theoretical section. The final model (Fig. 1) showed very good fit to the data, as judged by goodness of fit estimates. The chi-square test, χ^2^(1) = 0.28, *p* = .59, was insignificant. The GFI and AGFI were both above their desired levels (GFI = 1.00 and AGFI =1.00). The results for RMSEA and SRMR fell within range of acceptable values (RMSEA =0.00 and SRMR =0.005). The upper confidence limit for RMSEA was 0.09, and the RMSEA-based test of close fit also indicated good fit (*p* = .78).

The total *R* ^*2*^ was .40 for global sense of identity, .28 for integrative self-knowledge, .18 for reflection, .04 for imperviousness, and .11 for over-responsiveness. The significant indirect effects are presented in standardized form in Table 3.^5^ Considering the total effects of all constructs on global sense of identity, need for cognition exhibited the greatest influence (total effect = .43, *p* < .001), followed by over-responsiveness (total effect = − .39, *p* < .001) and integrative self-knowledge (total effect = .35, *p* < .001). After adjusting for indirect effects, the total effects of imperviousness and reflection on global sense of identity were determined to be insignificant (total effect = .02 and −.06, *ns*, respectively).

**Table 3:** Indirect Effects for the Final Structural Equation Model

Path	Dependent construct					
	REF		ISK		GSI	
	Effect	*SE*	Effect	*SE*	Effect	*SE*
NC → IP	.0484***	.0006	.0454***	.0004	-.0175*	.0002
NC → OR	–	–	.0996***	.0006	.0942***	.0003
NC → REF	–	–	.0302*	.0004	-.0275*	.0002
NC → ISK	–	–	–	–	.0821***	.0003
NC → IP → REF	–	–	.0050*	.0001	-.0045*	.0000
NC → IP → ISK	–	–	–	–	.0159***	.0001
NC → OR → ISK	–	–	–	–	.0348***	.0001
NC → REF → ISK	–	–	–	–	.0106*	.0001
NC → IP → REF → ISK	–	–	–	–	.0017*	.0000
REF → ISK	–	–	–	–	.0358*	.0061
IP → REF	–	–	-.0254*	.0279	.0232*	.0131
IP → ISK	–	–	–	–	-.0815***	.0220
IP → REF → ISK	–	–	–	–	-.0089*	.0052
OR → ISK	–	–	–	–	-.1046***	.0242

*NC* = need for cognition, REF = reflection, ISK = integrative self-knowledge, IP = imperviousness, OR = over-responsiveness, GSI = global sense of identity. Standardized coefficients are presented. ***p < .001, *p < .05

Given the observed sex differences (see Tables 1 and 2), a second set of analyses was conducted in which the invariance of path coefficients across the two gender groups was tested. A freely estimated model was compared to a model in which the relations between variables were set to be equal for women (*n* = 323) and men (*n* = 221). The chi-square difference test was used to determine whether these models were equivalent. When these two models were compared, there was a significant chi-square difference, Δχ^2^(Δdf = 15) = 61.89, *p* < .001. Thus, the relations between the variables were different for women and men.

**Fig. 2:**
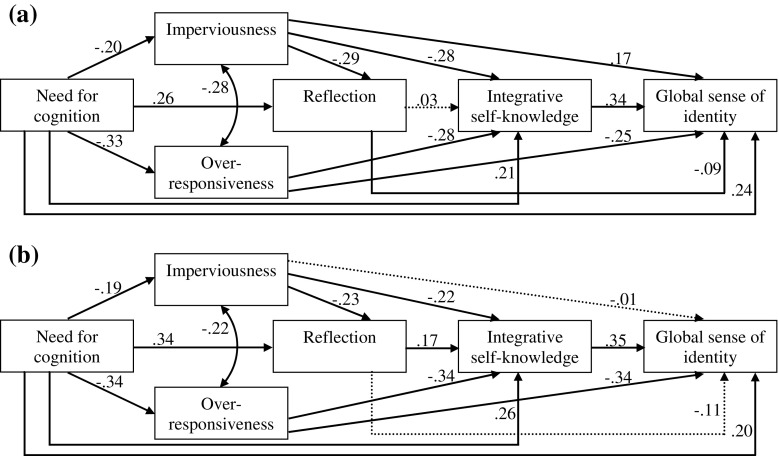
Structural models for women **a** and men **b**. Path coefficients are common metric standardized parameter estimates. Dotted lines indicate nonsignificant paths. All remaining paths were significant at the *p* < .05 level or better

The top panel of Fig. 2 shows the model for women depicting the pathways from need for cognition to global sense of identity, with modes of responding to discrepant information about the self, reflection, and integrative self-knowledge as potential mediators. The bottom panel shows the model for men. The total *R* ^*2*^ for prediction of global sense of identity was .42 for women and .38 for men.

For women, there were direct paths from each cognitive-motivational factor to global sense of identity (β range = −.09 to .34, *p* < .05). For men, there were no direct paths from imperviousness and reflection to global sense of identity (β = −.01 and β = −.11, *ns*, respectively). The majority of the significant direct paths were of considerably high-significance levels and in the expected direction. The one exception was the negative direct effect of reflection on women’s global sense of identity.

Integrative self-knowledge was the strongest direct predictor of global sense of identity for both genders (β = .34 and β = .35, *p* < .001 for women and men, respectively). Need for cognition directly (β = .21 and β = .26, *p* < .001 for women and men, respectively) and indirectly (total indirect effect = .16 and .22, *p* < .001 for women and men, respectively) predicted integrative self-knowledge through modes of coping and, for men only, reflection. Both the direct and total indirect effects of need for cognition were positive and stronger for males than for females (total effect = .37 and .48, *p* < .001 for women and men, respectively). For both genders, though more pronounced in males, need for cognition had its strongest indirect effect via over-responsiveness (indirect effect = .09 and .11, *p* < .001 for women and men, respectively). Imperviousness alone mediated the relation between need for cognition and integrative self-knowledge to a larger extent among women than men (indirect effect = .06 and .04, *p* < .01 for women and men, respectively). The indirect effects of need for cognition via reflection and both imperviousness and reflection in series reached significance only for men (indirect effect = .06, *p* = .003 and .01, *p* = .041, respectively). The effects of need for cognition on integrative self-knowledge via reflection did differ significantly between the gender groups (*t* = 2.08, *p* = .038). Over-responsiveness was the strongest direct predictor of integrative self-knowledge (β = −.28 and β = −.34, *p* < .001 for women and men, respectively), followed by imperviousness (β = −.28 and β = −.22, *p* < .001 for women and men, respectively). Among men, but not women, the indirect effect of imperviousness, operating through reflection was significant (indirect effect = − .04, *p* = .007), yielding a total effect of imperviousness on integrative self-knowledge of −.29 and −.26 for women and men, respectively (*p* < .001). These findings suggested that, in men, the diminishing influence of over-responsiveness on integrative self-knowledge was more pronounced (as compared to the influence of imperviousness), whereas in women the two effects were about the same size. Also only among men, reflection directly and positively predicted integrative self-knowledge (β = .17, *p* < .001); the difference between regression coefficients in men and women was marginally significant (*t* = 1.89, *p* = .059). The total *R* ^*2*^ for prediction of integrative self-knowledge was .25 for women and .36 for men.

Reflection directly and negatively influenced women’s global sense of identity (β = −.09, *p* = .042), while indirectly – operating via integrative self-knowledge – and positively affected men’s global sense of identity (indirect effect = .06, *p* = .004). The latter effect was marginally stronger for men when compared to women (*t* = 1.76, *p* = .078). For women as well as men, the direct and indirect effects of reflection were in opposite directions and canceled each other out, resulting in a non-significant total effect (total effect = − .08 and −.05, *ns* for women and men, respectively). For both genders, reflection was positively predicted by need for cognition (β = .26 and β = .34, *p* < .001 for women and men, respectively) and negatively by imperviousness (β = −.29 and β = −.23 *p* < .001 for women and men, respectively). Imperviousness also acted as a mediator in the relationship of need for cognition and reflection (indirect effect = .06 and .04, *p* < .01 for women and men, respectively). Overall, reflection was more closely related to need for cognition (total effect = .32 and .38, *p* < .001 for women and men, respectively) than to imperviousness, especially among men. The total *R* ^*2*^ for prediction of reflection was .17 for women and .21 for men.

For both genders, the two modes of responding to discrepant information about the self were negatively predicted by need for cognition. However, need for cognition was a more potent predictor of over-responsiveness (9.7 % and 14.0 % of variance explained for women and men, respectively) than of imperviousness (4.3 % and 3.4 % of variance explained for women and men, respectively). Imperviousness was positively and directly predictive for women’s global sense of identity (β = .17, *p* < .001), but not for men’s. The difference between regression coefficients in men and women was significant (*t* = 2.53, *p* = .012). Global sense of identity was influenced by imperviousness also indirectly through integrative self-knowledge (indirect effect = − .10 and −.08, *p* < .001 for women and men, respectively) and, among men only, through reflection and integrative self-knowledge operating in serial (indirect effect = − .01, *p* = .015). The total indirect effect was for both genders of the same magnitude of .07 (*p* < .01) and was negative, primarily because higher imperviousness reduced integrative self-knowledge, which led to lower sense of identity. The total effect of imperviousness on global sense of identity was opposite in direction, but failed to reach significance for both females and males (total effect = .09 and −.08, *ns*, respectively). Regardless the gender, over-responsiveness affected global sense of identity negatively directly (β = −.25 and β = −.34, *p* < .001 for women and men, respectively) and indirectly via integrative self-knowledge (indirect effect = − .10 and .12, *p* < .001 for women and men, respectively). Both direct and indirect effects of over-responsiveness were more pronounced in men that in women.

Need for cognition had both direct (β = .24 and β = .20, *p* < .001 for women and men, respectively) and indirect effects (indirect effect = .15 and .24, *p* < .001 for women and men, respectively) on global sense of identity. The indirect effect was marginally greater for men (*t* = 1.78, *p* = .075). The total effect of need for cognition on global sense of identity was .39 and .45 for women and men, respectively (*p* < .001). For both genders, need for cognition had the strongest indirect effect on global sense of identity via over-responsiveness acting as a single mediator (indirect effect = .08 and .11, *p* < .001 for women and men, respectively). Need for cognition also showed an indirect effect via integrative self-knowledge acting as the sole mediator (indirect effect = .07 and .09, *p* < .001 for women and men, respectively) as well as both over-responsiveness and integrative self knowledge (indirect effect = .03 and .04, *p* < .001 for women and men, respectively), and imperviousness and integrative self-knowledge (indirect effect = .02 and .01, *p* < .01 for women and men, respectively) as serial mediators. Only among men, reflection – acting in sequence with integrative self-knowledge – mediated the relationship of need for cognition and global sense of identity (indirect effect = .02, *p* < .009). Only among women, the positive effect of need for cognition on global sense of identity was suppressed by the significant negative indirect effect via imperviousness (indirect effect = − .03, *p* = .013). The path from need for cognition to global sense of identity through imperviousness was stronger for women when compared to men (*t* = 2.14, *p* = .033), whereas the path through reflection and integrative self-knowledge operating as serial mediators was stronger for men than women (*t* = 1.97, *p* = .050).

Overall, among the fourteen hypotheses proposed, ten were supported (H1, H2, H4, H5, H6, H8, H9, H10, H12, and H13) and two – concerning the effect of over-responsiveness on reflection (H7) and the effect of reflection on sense of identity (H11) – were not supported by the above results. The other two hypotheses were partially supported – the expected association between reflection and integrative self-knowledge (H3), and between imperviousness and sense of identity (H14) appeared to be conditional on gender.

## Discussion

One of the core ideas underlying many theories of personality is that construction of a stable and coherent identity is crucial part of healthy development (e.g., Freud 2005; Lecky 1945; Rogers 1959). As Erikson (1980) noted, the subjective experience of identity actually gives rise to a preconscious sense of personal well-being. Identity issues have been of considerable interest over the last decades due to the changing nature of the modern world and the need for new understandings of identity. The focus of this paper was to propose and test a model that addresses the potential role of cognitive-motivational factors in relation to sense of identity. The model was based upon the notion, well grounded in theoretical and empirical research, that a sense of identity emerges from cognitive processing of self-relevant information (e.g., Berzonsky 1991; Bosma and Kunnen 2001; Whitbourne 1986). Gender differences in relationships between cognitive-motivational factors and personal identity were also examined, although no predictions about the direction of the differences were made.

Most hypotheses concerning relationships among cognitive-motivational factors and their impact on personal identity were supported. Correlation analyses revealed, as expected and consistent with other studies (e.g., Berzonsky and Sullivan 1992; Njus and Johnson 2008), that active cognitive processing – especially efforts to integrate self-experience across time – was positively related to sense of identity. Correlations of reflection and senses of identity were more limited and less consistent. Reflection did correlate positively with sense of uniqueness, which is in line with the finding of Trapnell (1995) who showed that similarity ratings decreased with increasing reflection. Reflection was also positively correlated with sense of self-worth in the entire group, but not in women or men alone. In females only, an increase in reflection was associated with a decrease in sense of coherence. This was contrary to theoretical expectations, but consistent with studies indicating that reflection is negatively associated with self-concept clarity (Campbell et al. 1996; Johnson and Nozick 2011).

Growths of all specific identity senses and global sense of identity were consistently accompanied by a decrease in over-responsiveness to self-related discrepancies, as predicted from Whitbourne’s identity processing theory (Whitbourne et al. 2002). Imperviousness was largely unrelated to identity variables, but showed a positive association with sense of one’s own boundaries, suggesting that one’s resistance to acknowledge identity-discrepant experiences or incorporate new self-related information may be seen as an attempt to strengthen one’s sense of boundaries. Yet the patterns of associations differed by gender such that for women, higher imperviousness was also associated with stronger sense of uniqueness and global sense of identity, whereas for men no such relationships emerged. Given the crucial role of self-esteem in identity dynamics (e.g., Schwartz et al. 2005a; Vignoles et al. 2002), this result concurs somewhat with Skultety and Whitbourne (2004), who reported that identity assimilation was positively associated with self-esteem for women only.

The model describing the associations between cognitive-motivational factors and sense of personal identity was also verified by means of path analysis. Results were generally supportive of the theoretical model and consistent with correlation findings.

As expected, need for cognition proved to be a powerful positive predictor of global sense of identity. However, nearly half of its total effect was due to indirect effects. It also became apparent that need for cognition could have both positive and negative indirect effects on identity, mediated by different factors. On one hand, higher need for cognition was associated with higher integrative self-knowledge and lower over-responsiveness, which had positive effects on sense of identity. On the other hand, higher need for cognition predicted higher reflection and lower imperviousness, which in turn contributed to weaker sense of identity, but these effects were not strong enough to nullify the overall beneficial impact of need for cognition on global sense of identity.

Substantial impacts of over-responsiveness and integrative self-knowledge on global sense of identity were also evident. Consistent with what was expected, increased integrative self-knowledge was directly related to enhanced global sense of identity, while greater over-responsiveness was directly related to lowered sense of identity. Over-responsiveness was also found to predict integrative self-knowledge, with lower over-responsiveness predicting higher integrative self-knowledge.

Additionally, the model captured two conflicting effects of reflection on global sense of identity: heightened reflection directly contributed to weaker sense of identity, but also to increased integrative self-knowledge, which was associated with stronger sense of identity. Ultimately, because the direct and indirect effects canceled each other out, the total effect of reflection on global sense of identity was insignificant. Opposite effects were found for imperviousness as well. Higher imperviousness had beneficial impact on sense of identity, both directly and by reducing reflection. However, in both cases it resulted also in decreased integrative self-knowledge, thus indirectly lowering global sense of identity. Once again, the conflicting effects canceled each other out and produced a nonsignificant total effect of imperviousness.

In the final phase of the data analysis, the multi-sample analysis was used to determine whether there were meaningful gender differences in the relationships between variables. The models for women and men were rather alike, which was not surprising since the general model have demonstrated a very good model fit. However, some differences were observed.

For both genders, integrative self-knowledge, compared to the other constructs examined, had the highest direct effect on global sense of identity, followed by over-responsiveness and need for cognition. The magnitude of the latter effect was slightly (not significantly) higher in women than in men. The overall indirect relationship between need for cognition and global sense of identity was positive in both groups, but appeared to be marginally stronger for men. The main reason for this difference was that the positive need for cognition’s effects mediated by over-responsiveness and integrative self-knowledge were slightly greater for men compared to women. In addition, among women, the indirect path from need for cognition to imperviousness to global sense of identity was that high levels of need for cognition were associated with low levels of imperviousness, which in turn were associated with low levels of global sense of identity. The total effect of need for cognition on global sense of identity was numerically, but not significantly, greater in males. Over-responsiveness to self-related discrepancies was also found to be somewhat more relevant for men’s than for women’s global sense of identity. Both direct and indirect (through integrative self-knowledge) links between over-responsiveness and global sense of identity were apparently, although – again – not significantly, stronger for men than women.

The above results highlight need for cognition as a potent contributor to sense of personal identity, thus providing further support to the role of intrinsic cognitive motivation in self-understanding and self-regulation (e.g., Berzonsky and Sullivan 1992; Campbell et al. 1996; Cacioppo et al. 1996; Trapnell and Campbell 1999). An individual’s enjoyment of and motivation to engage in effortful cognitive processing enhances one’s self-reflective capacities, non-defensive reliance on internal referents, and teleological coherence, each of which, alone or in combination, impacts one’s sense of identity. Across the sexes, the detrimental effects appear to accompany excessive reliance on accommodation, though they might be more painful for men, possibly because women (at least in Western societies) are more likely to be encouraged to be attentive and responsive to others (Cross and Madson 1997; Li 2002; Madson and Trafimow 2001), and, as has been shown by some studies (e.g., Gurin et al. 1978; Itzhaky and Ribner 1999), they tend to be more externally oriented.

Furthermore, the results from the multi-sample analysis shed more light on the relationships between reflection, imperviousness to self-related discrepancies, and sense of identity. For women, it appeared that imperviousness had a direct beneficial effect, while reflection had a direct adverse effect on global sense of identity. For men these effects were only indirect and their direction was opposite. Imperviousness indirectly weakened men’s global sense of identity by reducing reflection and integrative self-knowledge, while reflection indirectly enhanced men’s global sense of identity by promoting integrative self-knowledge.

These findings may indicate that women and men differ in their use of assimilation in maintaining sense of identity. Although men were more likely to deal with discrepant information about themselves by minimizing or denying its significance, only for women such a way of coping provided a means of strengthening their sense of identity. Yet, this was accomplished somewhat at the expense of suppressing the reflective and integrative functions of the self; the same effect to which men were susceptible. Skultety and Whitbourne (2004), when commenting on the positive link of identity assimilation with self-esteem among women, elaborated on the “double standard” in society. They state that, because women’s value in our society is more closely tied to their appearance than is that of men, women’s identities may be more challenged by age-related changes in physical functioning and appearance. Thus, women benefit from identity assimilation more so than men do. Another way to understand the positive effect of imperviousness on women’s global sense of identity is in the context of the present findings that women had a weaker sense of their own boundaries than men, and that assimilative coping and sense of one’s own boundaries were positively related. Since the females’ self-concepts tend to be more relational and interdependent than those of men (e.g., Cross and Madson 1997; Madson and Trafimow 2001), assimilation (even if excessive) appears to be a mechanism that allows women to secure their fragile boundaries, and protect their sense of personal identity, albeit denial might not be the most optimal strategy.

As for the gender difference in the role of reflection, some research suggested that women may be higher than man in private self-consciousness (e.g., Alanazi 2001; Realo and Allik 1998; Scheier and Carver 1985). There is also some evidence that self-focus is more likely to affect women’s emotional experience (Flett et al. 1986; Mor and Winquist 2002), and that experimentally induced self-reflection is more likely to influence self-clarity for women than for men (Csank and Conway 2004). In the current study, contrary to that of Trapnell and Campbell (1999) and Zanon and Teixeira (2006), but in line with Luyckx et al.’s (2007) results, men reported engaging in reflection more than women did. Moreover, in men reflection facilitated the temporal integration of personal past, present, and future, resulting in a stronger sense of identity, whereas in women engaging in reflection appeared to decrease their sense of identity. Thus, although it is generally agreed that reflection contributes to psychological adjustment (e.g., Elliott and Coker 2008; Takano and Tanno 2009; Thomsen et al. 2011; Trapnell and Campbell 1999), it might be that for women particularly, reflection should not be considered purely adaptive. The fact that reflection and rumination are often found to correlate with each other (e.g., Luyckx et al. 2007; Takano and Tanno 2009), and that self-focus and negative affect tend to be more strongly related in female-dominated samples (Mor and Winquist 2002) add some support to this conclusion.

In sum, the data provide strong evidence for substantial contribution of cognitive-motivational factors to sense of personal identity. Taken together with previous research in this area, the findings add to a growing body of literature that empirically demonstrates the importance of active cognitive engagement in identity formation. Present results also indicate that certain kinds of cognitive processing, including imperviousness to self-related discrepancies and reflection, may have somewhat different implications for the two sexes.

The novelty of this study was its focus on the simultaneous effects of various cognitive-motivational factors, both adaptive and maladaptive. Structural equation modeling allowed to go beyond examining bivariate relations and provided insight into the way that observed correlations between cognitive-motivational and identity variables can be decomposed into direct and indirect effects. This approach proved especially valuable in the case of reflection, whose adaptive nature remains controversial (Şimşek et al. 2013; Takano and Tanno 2009). Furthermore, since most identity researchers have employed Marcia’s (1966) identity-status paradigm and its extensions, within which the subjective experiential aspect of identity has tended to be neglected (see Blasi and Glodis 1995; Schwartz 2005), it is worth noting that the proposed conceptualization of identity emphasizes a person’s self-experience and self-understanding, and relates to the phenomenological perspective.

## Limitations and Future Directions

There are a few limitations to the current study. First, the study included Polish young adults only. The relative homogeneity of the sample provided a more rigorous test of hypotheses and allowed fewer confounding variables to interfere, but limited generalization of findings to other demographic and cultural contexts. It is possible that the identity process itself operates differently and relative salience of sources of self-knowledge varies by social characteristic such as age and ethnicity. There is, for example, evidence that individuals from primarily collectivist cultures (or those with an interdependent self-construal) may be more likely than those from primarily individualistic cultures (or those with an independent self-construal) to perceive social sources (i.e., reflected appraisal, social comparison) as more important to self-knowledge than introspection (Suh 2007; Tafarodi et al. 2004). Certainly, a replication of the present results with more diverse samples is warranted.

Another limitation could be the method used to assess the two modes of responding to discrepant information about the self. Although the CWD scale demonstrated an adequate fit to the factor structure, the reliability was not as high as desired or expected for either subscale. The relatively low internal consistency, especially of the over-responsiveness subscale, which may have been partly due to the small number of items in the subscale, calls for caution of interpretation and further evaluation of the usefulness of the CWD scale.

Also, this study did not measure many of the personality variables potentially impacting the cognition–identity link (see, Sedikides and Skowronski 1995) as well as other psychological and social factors which may facilitate or impede the development and maintenance of a sense of identity. It is worth noting, that although the overall fit of the model was good, it left a significant amount of variance still to be explained. This suggests that there is important variability in sense of identity that is not captured by the cognitive-motivational mechanisms examined in this study, and provides support for the further investigation into identity formation and dynamics.

## Footnotes

1 A version of MQI modified by its author was used. The subscales of specificity and stability were extended in order to improve their internal consistencies and widen the range of thoughts, feelings or actions the items in these subscales refer to.

2 For all multi-item measures, person-mean substitution was used to replace missing values for participants missing up to 20 % items in the (sub)scale. For participants missing a greater number of items, the score on that (sub)scale was not calculated.

3 The data were screened for outliers using the so-called boxplot outlier labeling rule. That rule declares observations as outliers if they lie outside the interval ((Q1 - g(Q3 – Q1), Q3 + g(Q3 – Q1)), where g = 2.2 (Hoaglin and Iglewicz 1987). Identified outliers (nine altogether) were removed only from the particular measure; that is, individuals whose scores were outliers on one measure were not removed on other measures in the analyses. The results, however, were not sensitive to the removal of outliers.

4 Due to the relatively wide age range of participants in this study, the bivariate correlations between age and the other measures were also examined. The analyses for the total sample revealed only weak relationships of age with need for cognition (*r* = .10, *p* = .024), reflection (*r* = .10, *p* = .017), integrative self-knowledge (*r* = .09, *p* = .030), and sense of coherence (*r* = .12, *p* = .005). However, given the intercorrelations among cognitive-motivational and identity variables (see Table 2), the interpretation of these relationships should be done with care. For example, the positive association between age and sense of coherence did not hold when controlling for need for cognition and integrative self-knowledge (β = .07, *p* > .05). Full results are available on request.

5 The Sobel tests (Sobel 1982) and the procedures described by Barron and Kenny (1986) were used to evaluate statistical significance of indirect effects (see also Taylor et al. 2008).

## References

[CR1] Alanazi FM (2001). The revised self-consciousness scale: An assessment of factor structure, reliability, and gender differences in Saudi Arabia. Social Behavior and Personality: An International Journal.

[CR2] Alloy LB, Abramson LY (1979). Judgment of contingency in depressed and nondepressed students: sadder but wiser?. Journal of Experimental Psychology: General.

[CR3] Anderson EM, Bohon LM, Berrigan LP (1996). Factor structure of the Private Self-Consciousness Scale. Journal of Personality Assessment.

[CR4] Barron RM, Kenny DA (1986). The moderator-mediator variable distinction in social psychological research: conceptual, strategic, and statistical considerations. Journal of Personality and Social Psychology.

[CR5] Berzonsky MD (1991). *A process view of identity formation and maintenance*. paper presented at the biennial meeting of the Society For Research in child development.

[CR6] Berzonsky MD, Luyckx K (2008). Identity styles, self-reflective cognition, and identity processes: A study of adaptive and maladaptive dimensions of self-analysis. Identity: An International Journal of Theory and Research.

[CR7] Berzonsky MD, Sullivan C (1992). Social-cognitive aspects of identity style: need for cognition, experiential openness, and introspection. Journal of Adolescent Research.

[CR8] Blasi A, Glodis K (1995). The development of identity: A critical analysis from the perspective of the self as subject. Developmental Review.

[CR9] Bosma HA, Kunnen ES (2001). Determinants and mechanisms in ego identity development: A review and synthesis. Developmental Review.

[CR10] Breakwell GM (1993). Social representations and social identity. Papers on Social Representations.

[CR11] Burnkrant RE, Page TJ (1984). A modification of the fenigstein, scheier, and buss self-consciousness Scales. Journal of Personality Assessment.

[CR12] Cacioppo JT, Petty RE (1982). The need for cognition. Journal of Personality and Social Psychology.

[CR13] Cacioppo JT, Petty RE, Morris KJ (1983). Effects of need for cognition on message evaluation, recall, and persuasion. Journal of Personality and Social Psychology.

[CR14] Cacioppo JT, Petty RE, Kao CF, Rodriguez R (1986). Central and peripheral routes to persuasion: An individual difference perspective. Journal of Personality and Social Psychology.

[CR15] Cacioppo JT, Petty RE, Feinstein JA, Jarvis WBG (1996). Dispositional differences in cognitive motivation: the life and times of individuals varying in need for cognition. Psychological Bulletin.

[CR16] Campbell JD, Trapnell PD, Heine SJ, Katz IM, Lavallee LF, Lehman DR (1996). Self-concept clarity: measurement, personality correlates, and cultural boundaries. Journal of Personality and Social Psychology.

[CR17] Cross SE, Madson L (1997). Models of the self: self-construals and gender. Psychological Bulletin.

[CR18] Csank PAR, Conway M (2004). Engaging in self-reflection changes self-concept clarity: On differences between women and men, and low- and high-clarity individuals. Sex Roles.

[CR19] Elliott I, Coker S (2008). Independent self-construal, self-reflection, and self-rumination: A path model for predicting happiness. Australian Journal of Psychology.

[CR20] Epstein, S. (2003). Cognitive-experiential self-theory of personality. In T. Millon &M. J. Lerner (Eds.), *Handbook of psychology*: *Vol. 5. Personality and social psychology* (pp. 159–184). Hoboken: Wiley.

[CR21] Erikson EH (1968). *Identity*: *Youth and crisis*.

[CR22] Erikson EH (1980). Identity and the life cycle.

[CR23] Fenigstein A, Scheier MF, Buss AH (1975). Public and private self-consciousness: assessment and theory. Journal of Consulting and Clinical Psychology.

[CR24] Fleckhammer, L. (2004). *Insight into the self-absorption paradox*: *The development of a multi-faceted model of self-conscious ruminative and reflective thought* (Doctoral Dissertation). Retrieved from http://researchbank.swinburne.edu.au/

[CR25] Fleischhauer M, Enge S, Brocke B, Ullrich J, Strobel A, Strobel A (2010). Same or different? clarifying the relationship of need for cognition to personality and intelligence. Personality and Social Psychology Bulletin.

[CR26] Fletcher GJO, Danilovics P, Fernandez G, Peterson D, Reeder GD (1986). Attributional complexity: An individual differences measure. Journal of Personality and Social Psychology.

[CR27] Flett GL, Boase P, McAndrews MP, Pliner P, Blankstein KR (1986). Affect intensity and the appraisal of emotion. Journal of Research in Personality.

[CR28] Freud S (2005). *Poza zasadą przyjemności* [beyond the pleasure principle].

[CR29] Ghorbani N, Watson PJ, Krauss SW, Davison HK, Bing MN (2004). Private self-consciousness factors: relationships with need for cognition, locus of control, and obsessive thinking in Iran and the United States. Journal of Social Psychology.

[CR30] Ghorbani N, Watson PJ, Hargis MB (2008). Integrative self-knowledge scale: correlations and incremental validity of a cross-cultural measure developed in Iran and the United States. Journal of Psychology.

[CR31] Ghorbani N, Watson PJ, Zarehi J, Shamohammadi K (2010). Muslim extrinsic cultural religious orientation and identity: relationships with social and personal adjustment in Iran. Journal of Beliefs and Values.

[CR32] Ghorbani N, Watson PJ, Chen Z, Dover H (2013). Varieties of openness in Tehran and Qom: psychological and religious parallels of faith and intellect-oriented Islamic religious reflection. Mental Health, Religion & Culture.

[CR33] Ghorbani N, Watson PJ, Salimian M, Chen Z (2013). Shame and guilt: relationships of test of self-conscious affect measures with psychological adjustment and gender differences in Iran. Interpesona: An International Journal on Personal Relationships.

[CR34] Goth K, Foelsch P, Schlüter-Müller S, Birkhölzer M, Jung E, Pick O, Schmeck K (2012). Assessment of identity development and identity diffusion in adolescence – theoretical basis and psychometric properties of the self-report questionnaire AIDA. Child and Adolescent Psychiatry and Mental Health.

[CR35] Grotevant HD (1987). Toward a process model of identity formation. Journal of Adolescent Research.

[CR36] Gurin P, Gurin G, Morrison BM (1978). Personal and ideological aspects of internal and external control. Social Psychology.

[CR37] Haddock G, Maio GR, Arnold K, Huskinson TL (2008). Should persuasion be affective or cognitive? the moderating effects of need for affect and need for cognition. Personality and Social Psychology Bulletin.

[CR38] Harrington R, Loffredo DA (2010). Insight, rumination, and self-reflection as predictors of well-being. The Journal of Psychology.

[CR39] Haugtvedt CP, Petty RE (1992). Personality and persuasion: need for cognition moderates the persistence and resistance of attitude changes. Journal of Personality and Social Psychology.

[CR40] Hilgard ER (1949). Human motives and the concept of the self. American Psychologist.

[CR41] Hoaglin DC, Iglewicz B (1987). Fine tuning some resistant rules for outlier labeling. Journal of American Statistical Association.

[CR42] Inhelder B, Piaget J (1958). The growth of logical thinking from childhood to adolescence.

[CR43] Itzhaky H, Ribner DS (1999). Gender, values and the work place: considerations for immigrant acculturation. International Social Work.

[CR44] Johnson EA, Nozick KJ (2011). Personality, adjustment, and identity style influences on stability in identity and self-concept during the transition to university. Identity: An International Journal of Theory and Research.

[CR45] Joireman JA, Parrott L, Hammersla J (2002). Empathy and the self-absorption paradox: support for the distinction between self-rumination and self-reflection. Self and Identity.

[CR46] Kerpelman JL, Pittman JF, Lamke LK (1997). Toward a microprocess perspective on adolescent identity development: An identity control theory approach. Journal of Adolescent Research.

[CR47] Klaczynski PA, Fauth JM, Swanger A (1998). Adolescent identity: rational vs. experiential processing, formal operations, and critical thinking beliefs. Journal of Youth and Adolescence.

[CR48] Kline RB (1998). Principles and practice of structural equation modeling.

[CR49] Kroger J (2002). Identity processes and contents through the years of late adulthood. Identity: An International Journal of Theory and Research.

[CR50] Kunnen ES, Sappa V, van Geert PLC, Bonica L (2008). The shapes of commitment development in emerging adulthood. Journal of Adult Development.

[CR51] Lecky P (1945). *Self-consistency*: *A theory of personality*.

[CR52] Li HZ (2002). Culture, gender and self-close-other(s) connectedness in Canadian and Chinese samples. European Journal of Social Psychology.

[CR53] Luyckx K, Soenens B, Berzonsky MD, Smits I, Goossens L, Vansteenkiste M (2007). Information-oriented identity processing, identity consolidation, and well-being: the moderating role of autonomy, self-reflection, and self-rumination. Personality and Individual Differences.

[CR54] Luyckx K, Schwartz SJ, Berzonsky MD, Soenens B, Vansteenkiste M, Smits I, Goossens L (2008). Capturing ruminative exploration: extending the four-dimensional model of identity formation in late adolescence. Journal of Research in Personality.

[CR55] Madson L, Trafimow D (2001). Gender comparisons in the private, collective, and allocentric selves. The Journal of Social Psychology.

[CR56] Marcia JE (1966). Development and validaton of ego-identity status. Journal of Personality and Social Psychology.

[CR57] Martin AJ, Debus RL (1999). Alternative factor structure for the revised self-consciousness scale. Journal of Personality Assessment.

[CR58] Maruszewski T (2010). Pamięć autobiograficzna i tożsamość [autobiographical memory and identity]. Czasopismo Psychologiczne.

[CR59] Matusz PJ, Traczyk J, Gąsiorowska A (2011). Kwestionariusz potrzeby poznania – konstrukcja i weryfikacja empiryczna narzędzia mierzącego motywację poznawczą [need for cognition questionnaire – construction and empirical verification of the scale measuring cognitive motivation]. Psychologia Społeczna.

[CR60] McAdams DP (1996). Personality, modernity, and the storied self: A contemporary framework for studying persons. Psychological Inquiry.

[CR61] McLean KC (2008). The emergence of narrative identity. Social and Personality Psychology Compass.

[CR62] Mittal B, Balasubramanian SK (1987). Testing the dimensionality of the self-consciousness Scales. Journal of Personality Assessment.

[CR63] Mor N, Winquist J (2002). Self-focused attention and negative affect: A meta-analysis. Psychological Bulletin.

[CR64] Neimeyer GJ, Metzler AE, Neisser U, Fivush R (1994). Personal identity and autobiographical recall. *The remembering self*: *Construction and accuracy in the self-narrative. Emory symposia in cognition*.

[CR65] Njus D, Johnson DR (2008). Need for cognition as a predictor of psychosocial identity development. The Journal of Psychology: Interdisciplinary and Applied.

[CR66] Oleś PK, Oleś PK, Batory A (2008). O różnych rodzajach tożsamości oraz ich stałości i zmianie [the different kinds of identities and their constancy, and change]. *Tożsamość i jej przemiany a kultura* [the identity and its transformation, and culture].

[CR67] Piaget J (1977). The development of thought equilibrium of cognitive structures.

[CR68] Pilarska A, Paluchowski WJ, Bujacz A, Haładziński P, Kaczmarek L (2012). Wielowymiarowy kwestionariusz Tożsamości [multidimensional questionnaire of identity]. *Nowoczesne metody badawcze w psychologii* [modern research methods in psychology].

[CR69] Pilarska A (2014). Self-construal as a mediator between identity structure and subjective well-being. Current Psychology.

[CR70] Pilarska, A. (2016). Psychometric properties and initial validation of the Polish translation of the Integrative Self-Knowledge Scale. *Polskie Forum Psychologiczne*, in press.

[CR71] Pilarska A, Suchańska A (2013). Strukturalne właściwości koncepcji siebie a poczucie tożsamości. fakty i artefakty w pomiarze spójności i złożoności koncepcji siebie [the relationship between structural aspects of self-concept and personal identity. facts and artifacts in the measurement of self-concept complexity and coherence]. Studia Psychologiczne.

[CR72] Pilarska A, Suchańska A (2013). *Złożoność Ja i zróżnicowanie Ja*: *pomiar i psychologiczne korelaty* [self-complexity and self-concept differentiation: measurement and psychological correlates].

[CR73] Polverino, A. M. (2010). Examining the roles of identity processing styles and self-perceptions of aging on well-being in later life. *ETD Collection for Fordham University.* Paper AAI3407467. Retrieved from http://fordham.bepress.com/dissertations/

[CR74] Realo A, Allik J (1998). The Estonian self-consciousness scale and its relation to the five-factor model of personality. Journal of Personality Assessment.

[CR75] Rogers C, Koch S (1959). A theory of therapy, personality and interpersonal relationships as developed in the client-centered framework. *Psychology*: *A study of a science*.

[CR76] Rowe, I. (1980). *Ego identity status*, *formal operations and moral development* (Master’s thesis, Simon Fraser University). Retrieved from http://www.researchgate.net/10.1007/BF02087928 24318013

[CR77] Ruiter RAC, Verplanken B, De Cremer D, Kok G (2004). Danger and fear control in response to fear appeals: the role of need for cognition. Basic and Applied Social Psychology.

[CR78] Sadowski CJ, Cogburn HE (1997). Need for cognition in the big-five factor structure. The Journal of Psychology.

[CR79] Scheier MF, Carver CS (1985). The self-consciousness scale: A revised version for use with general populations. Journal of Applied Social Psychology.

[CR80] Schwartz SJ (2005). A new identity for identity research: recommendations for expanding and refocusing the identity literature. Journal of Adolescent Research.

[CR81] Schwartz SJ, Côté JE, Arnett JJ (2005). Identity and agency in emerging adulthood: two developmental routes in the individualization process. Youth & Society.

[CR82] Schwartz SJ, Kurtines WM, Montgomery MJ (2005). A comparison of two approaches for facilitating identity exploration processes in emerging adults: An exploratory study. Journal of Adolescent Research.

[CR83] Sedikides C, Skowronski JJ (1995). On the sources of self-knowledge – the perceived primacy of self-reflection. Journal of Social and Clinical Psychology.

[CR84] Şimşek ÖF, Ceylandağ AE, Akcan G (2013). The need for absolute truth and self-rumination as basic suppressors in the relationship between private self-consciousness and mental health. The Journal of General Psychology.

[CR85] Singer JA (2004). Narrative identity and meaning making across the adult lifespan: An introduction. Journal of Personality.

[CR86] Skultety KM, Whitbourne SK (2004). Gender differences in identity processes and self-esteem in middle and later adulthood. Journal of Women and Aging.

[CR87] Sneed JR, Whitbourne SK (2003). Identity processing and self-consciousness in middle and later adulthood. Journal of Gerontology: Psychological Sciences and Social Sciences.

[CR88] Sobel ME (1982). Asymptotic confidence intervals for indirect effects in structural equation models. Sociological Methodology.

[CR89] Sokolik M (1996). *Psychoanaliza i Ja* [psychoanalysis and self].

[CR90] Suh EM (2007). Downsides of an overly context-sensitive self: implications from the culture and subjective well-being research. Journal of Personality.

[CR91] Tafarodi RW, Lo C, Yamaguchi S, Lee W, Katsura H (2004). The inner self in three countries. Journal of Cross-Cultural Psychology.

[CR92] Tahmasb AR, Ghorbani N, Watson PJ (2008). Relationships between self- and peer-reported integrative self-knowledge and the big five factors in Iran. Current Psychology.

[CR93] Takano K, Tanno Y (2009). Self-rumination, self-reflection, and depression: self-rumination counteracts the adaptive effect of self-reflection. Behaviour Research and Therapy.

[CR94] Taylor AB, MacKinnon DP, Tein J (2008). Tests of the three-path mediated effect. Organizational Research Methods.

[CR95] Thompson MM, Zanna MP, Griffin DW, Petty RE, Krosnick JA (1995). Let’s not be indifferent about (attitudinal) ambivalence. *Attitude strength*: *Antecedents and consequences*.

[CR96] Thomsen DK, Tønnesvang J, Schnieber A, Olesen MH (2011). Do people ruminate because they haven’t digested their goals? the relations of rumination and reflection to goal internalization and ambivalence. Motivation and Emotion.

[CR97] Trapnell, P. D. (1995). *Self-consciousness and the five factor model of personality*: *Distinguishing rumination from reflection* (Doctoral dissertation, University of British Columbia). Retrieved from https://circle.ubc.ca/10.1037//0022-3514.76.2.284 10074710

[CR98] Trapnell, P. D. (1997). RRQ Shortforms. Unpublished instrument. Retrieved from http://www.paultrapnell.com/measures/RRQshortforms.rtf

[CR99] Trapnell PD, Campbell JD (1999). Private self-consciousness and the five-factor model of personality: distinguishing rumination from reflection. Journal of Personality and Social Psychology.

[CR100] Trzebińska E (2002). Automatic and controlled processing in self-understanding. Polish Psychological Bulletin.

[CR101] Verplanken B, Hazenberg PT, Palenewen GT (1992). Need for cognition and external information search effort. Journal of Research in Personality.

[CR102] Vignoles VL, Chryssochoou X, Breakwell GM (2002). Evaluating models of identity motivation: self-esteem is not the whole story. Self and Identity.

[CR103] Vignoles VL, Regalia C, Manzi C, Golledge J, Scabini E (2006). Beyond self-esteem: influence of multiple motives on identity construction. Journal of Personality and Social Psychology.

[CR104] Westerhof GJ, Whitbourne SK, Freeman GP (2012). The aging self in a cultural context: the relation of conceptions of aging to identity processes and self-esteem in the United States and The Netherlands. Journals of Gerontology Series B: Psychological Sciences and Social Sciences.

[CR105] Whitbourne SK (1986). *The me I know*: *A study of adult identity*.

[CR106] Whitbourne SK, Magai C, McFadden SH (1996). Psychosocial perspectives on emotions: the role of identity in the aging process. *Handbook of emotion*, *adult development*, *and aging*.

[CR107] Whitbourne SK, Collins KJ (1998). Identity processes and perceptions of physical functioning in adults: theoretical and clinical implications. Psychotherapy.

[CR108] Whitbourne SK, Sneed JR, Skultety KM (2002). Identity processes in adulthood: theoretical and methodological challenges. Identity: An International Journal of Theory and Research.

[CR109] Zanon C, Teixeira MAP (2006). Adaptação do Questionário de Ruminação e Reflexão (QRR) para estudantes universitários brasileiros [adaptation of the rumination and reflection questionnaire (RRQ) to Brazilian university students]. Interação Em Psicologia.

